# Supplementary material for: From dawn till dusk: Time-adaptive bayesian optimization for neurostimulation
Source: PLoS Comput Biol. 2023 Dec 13;19(12):e1011674. doi: 10.1371/journal.pcbi.1011674 (PMC10718444; doi:10.1371/journal.pcbi.1011674)
Supplement: S1 Fig — Panels A, C and E illustrate the tracking performance of the TV-BayesOpt algorithm (blue dots) at locating the true optimal phase value (black dots) for population desynchronization when no noise, Gaussian distributed noise with zero mean and a 0.75 standard deviation or Gaussian distributed noise with zero mean and a 1.5 standard deviation was added to the simulated optimum trajectory. Panels B, D and F illustrate the associated average regret for the TV-BayesOpt algorithm at tracking the true optimum phase value in comparison to when static BayesOpt was implemented alone for tracking the optimum trajectory in their associated Panels A, C and E. (DOCX) [file pcbi.1011674.s001.docx]

**S1 Fig – TV-BayesOpt algorithm performance in presence of noise**


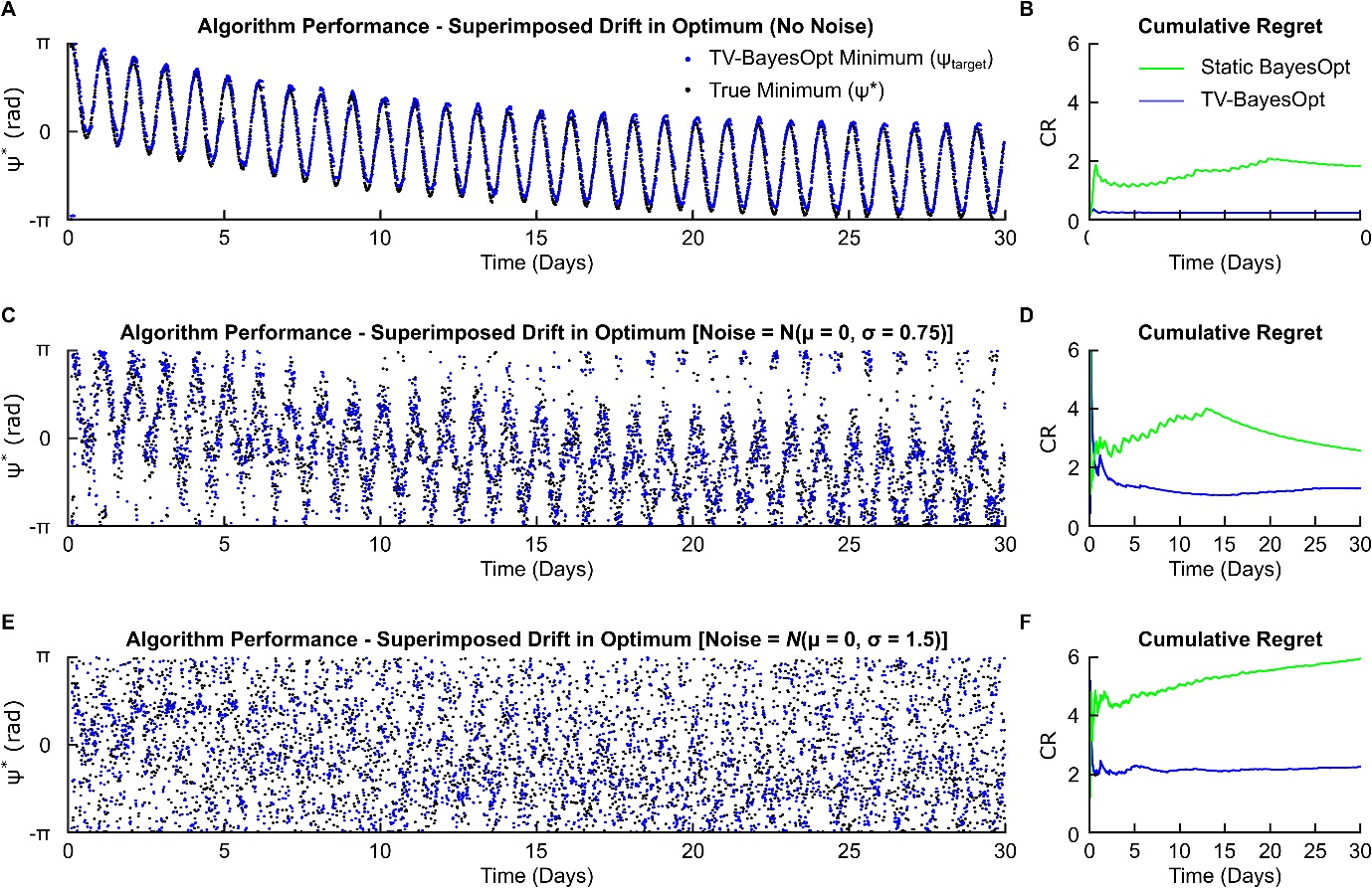


**S1 Fig: TV-BayesOpt algorithm performance for tracking a superimposed (gradual and periodic) drift in the optimal stimulation phase for phase-locked stimulation,** $\boldsymbol{\psi}^{\mathbf{*}}$**, in the presence of noise.** Panels A, C and E illustrate the tracking performance of the TV-BayesOpt algorithm (blue dots) at locating the true optimal phase value (black dots) for population desynchronization when no noise, Gaussian distributed noise with zero mean and a 0.75 standard deviation or Gaussian distributed noise with zero mean and a 1.5 standard deviation was added to the simulated optimum trajectory. Panels B, D and F illustrate the associated average regret for the TV-BayesOpt algorithm at tracking the true optimum phase value in comparison to when static BayesOpt was implemented alone for tracking the optimum trajectory in their associated Panels A, C and E.
