# Supplementary material for: From dawn till dusk: Time-adaptive bayesian optimization for neurostimulation
Source: PLoS Comput Biol. 2023 Dec 13;19(12):e1011674. doi: 10.1371/journal.pcbi.1011674 (PMC10718444; doi:10.1371/journal.pcbi.1011674)
Supplement: S2 Fig — Panels A, C, E and G illustrate the tracking performance of the TV-BayesOpt algorithm (blue dots) at locating the true optimal phase value (black dots) for population desynchronization when the Kuramoto model oscillator frequency was centered at 8 or 23 Hz. The frequency of oscillators in the underlying population were selected from a Gaussian distribution centered around each frequency. Panel C, E and G illustrate that increasing distribution of the oscillator natural frequencies, the size of the population or the natural frequency of the oscillator population does not affect algorithm performance at tracking the optimum stimulation phase since the shape of the objective is consistent across these parameters. Panels B, D, F and H illustrate the associated average regret for the TV-BayesOpt algorithm at tracking the true optimum phase value in comparison to when static BayesOpt was implemented alone for tracking the optimum trajectory in their associated panels A, C, E and G. (DOCX) [file pcbi.1011674.s002.docx]

**S2 Fig – TV-BayesOpt algorithm performance for varying Kuramoto model parameter values.**


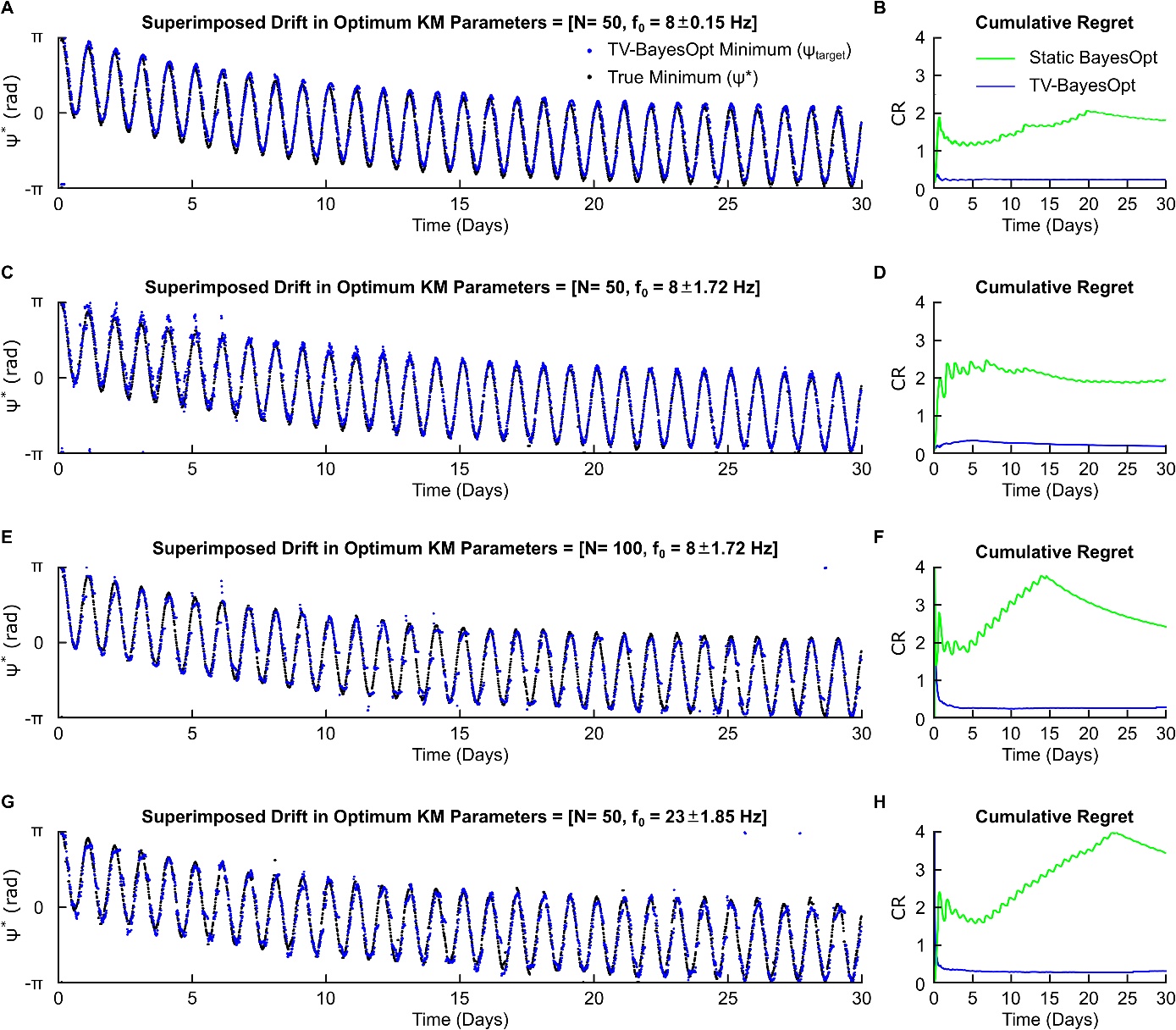


**S2 Fig: TV-BayesOpt algorithm performance for tracking a superimposed (gradual and periodic) drift in the optimal stimulation phase for phase-locked stimulation,** $\boldsymbol{\psi}^{\mathbf{*}}$**, for varying Kuramoto model parameter values.** Panels A, C, E and G illustrate the tracking performance of the TV-BayesOpt algorithm (blue dots) at locating the true optimal phase value (black dots) for population desynchronization when the Kuramoto model oscillator frequency was centered at 8 or 23 Hz. The frequency of oscillators in the underlying population were selected from a Gaussian distribution centered around each frequency. Panel C, E and G illustrate that increasing distribution of the oscillator natural frequencies, the size of the population or the natural frequency of the oscillator population does not affect algorithm performance at tracking the optimum stimulation phase since the shape of the objective is consistent across these parameters. Panels B, D, F and H illustrate the associated average regret for the TV-BayesOpt algorithm at tracking the true optimum phase value in comparison to when static BayesOpt was implemented alone for tracking the optimum trajectory in their associated panels A, C, E and G.
