# Supplementary material for: From dawn till dusk: Time-adaptive bayesian optimization for neurostimulation
Source: PLoS Comput Biol. 2023 Dec 13;19(12):e1011674. doi: 10.1371/journal.pcbi.1011674 (PMC10718444; doi:10.1371/journal.pcbi.1011674)
Supplement: Attachment — Submitted filename: TV_BayesOpt_Reviewer_Reviewer_Responses.pdf [file pcbi.1011674.s003.pdf]

PLOS Computational Biology

Manuscript reference – PCOMPBIOL-D-23-00902

From Dawn till Dusk: Time-Adaptive Bayesian Optimization for Neurostimulation

Response to Reviewers' Comments

The authors would like to thank the reviewers for their insightful comments on the paper. A response to each of the reviewers' comments is provided below, with reviewers' original comments given in italics. Updates to the manuscript are identified in each response below and are highlighted in the revised manuscript as blue, underlined text.

Reviewer 1

Comment 1:

The proposed approach is studied in the context of parametrization of the Kuramoto model which is commonly workhorse in phase-locked deep brain simulation for treatment of oscillopathies. To recap, the Kuramoto model is parametrized with an intrinsic frequency parameter/parameters describing the oscillation frequency of the N coupled neurons, coupling term, intensity of the stimulation and a standard deviation of population oscillators from the natural frequency. The manuscript has summarized the fixed values of the Kuramoto model parameters adopted in all experiments where only the timing (i.e. phase of the stimulation) is inferred with Bayesian optimization. The choice of the Kuramoto model is a very well-motivated but additional background information on practical uncertainty associated with the hyperparameters (in particular the natural frequency and the stimulation intensity) would be beneficial.

Comment 1 Response:

We thank the reviewer for this suggestion. Additional information on the model hyperparameters has now been added to the Methods Section 2.2. of the revised manuscript. The following text has been added between lines 121 – 137 to provide additional background on the selection of the natural frequency of the Kuramoto model:

"In a variety of neurological disorders, pathologically increased neuronal synchrony, reflected as an increase in power within a specific frequency band, is observed to correlate with disease

symptom severity (15–18). In this specific case, the Kuramoto Model represents a population of neurons as N coupled oscillators that oscillate about some center frequency, ω. To mimic synchronous activity associated with a specific disease pathology, the value of ω is selected to lie within a frequency band that is observed to correlate with the severity of the associated disease's symptoms. In this study, to model synchronous oscillatory activity characteristic of Essential Tremor, where tremor is observed to occur between 4 – 12 Hz, ω was selected as 8 Hz (41). The frequency of each oscillator simulated in the population was selected from a Gaussian distribution centered around 8 Hz with a standard deviation of 0.0075. This resulted in a distribution of oscillator frequencies in the population between 8 ± 0.15 Hz. In a practical application, ω would need to be identified in a patient-specific manner. This can be done by using accelerometer recordings from tremor patients to determine the patient specific average tremor frequency and associated frequency variability. Alternatively, for other neurological disorders ω may be identified using non-invasive and invasive neural biomarkers which can be derived using electroencephalogram (EEG) and local field potential (LFP) recordings."

We envision this algorithm to be used for oscillopathies where a clear rhythm can be identified in either peripheral or central signals. We enclose exemplary simulated power spectral densities in Figure 1. The left panel illustrates the mean-phase coherence of a synchronized (blue line) and a desynchronized (orange line) population of Kuramoto oscillators with natural frequencies 8 ± 0.15 Hz. The population coupling value γ was set to zero for the desynchronized population to prevent synchronization, in comparison to 0.8 for the synchronized population. A LFP signal was simulated for each population by convolving the summation of the population spike trains (where a spike was represented by an oscillator completing a full revolution from 0 to 2π rads) with a 25 ms width and 30 mV amplitude Gaussian. The corresponding power spectral density plots for the synchronized and desynchronized populations are illustrated in the right panel of Figure 1. Increased power is observed at the 8 ± 0.15 Hz natural frequency for the synchronized population and not for the desynchronized population. When there is a clear rhythm (increased power at a specific frequency band) the objective function (the ARC) is well-defined. If there is no clear rhythm, then no oscillopathy is present and the rhythmic objective function (the ARC) is not defined.

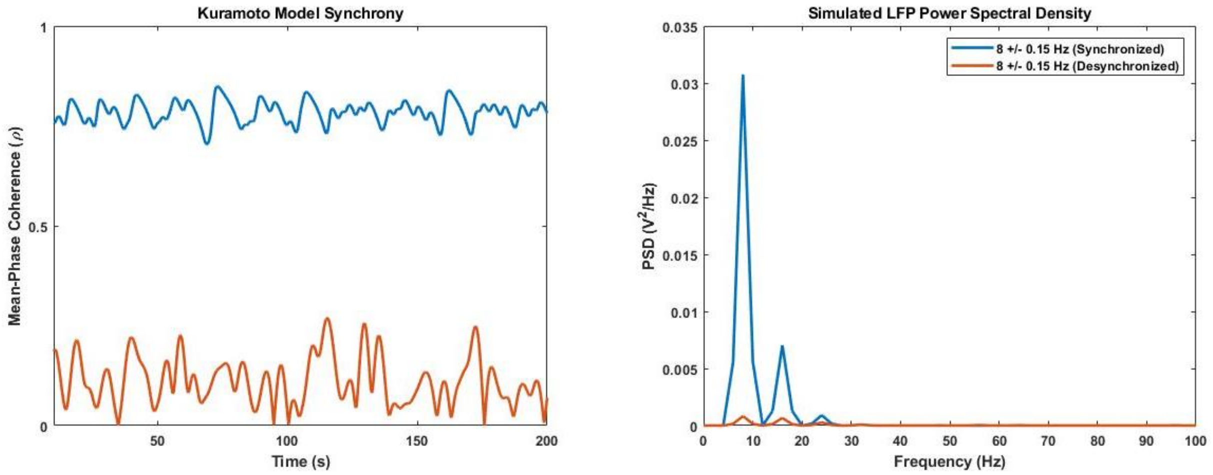

Figure 1: Example Simulated LFP Power Spectral Density for Synchronized and Desynchronized Kuramoto Oscillator Populations. Left panel illustrates the mean-phase coherence for a synchronized (blue line) and desynchronized (orange line) population whose natural oscillator frequency is 8 ± 0.15 Hz. Right panel illustrates the corresponding PSD for each population LFP signal where the LFP was simulated by convolving the summation of the population spike trains with a 25 ms width and 30 mV amplitude Gaussian.

It should be noted that increasing the variation on the distribution of oscillator frequencies in the population from 8 ± 0.15 Hz to 8 ± 1.72 Hz does not impact the algorithm performance. This is illustrated below in Figure 2 where the algorithm performance from the main manuscript at tracking a superimposed (gradual and periodic) drift in the optimal stimulation phase value , Figure 2.A, B, is compared to the scenario where the distribution of oscillator frequencies is increased to 8 ± 1.72 Hz, Figure 2.C, D.

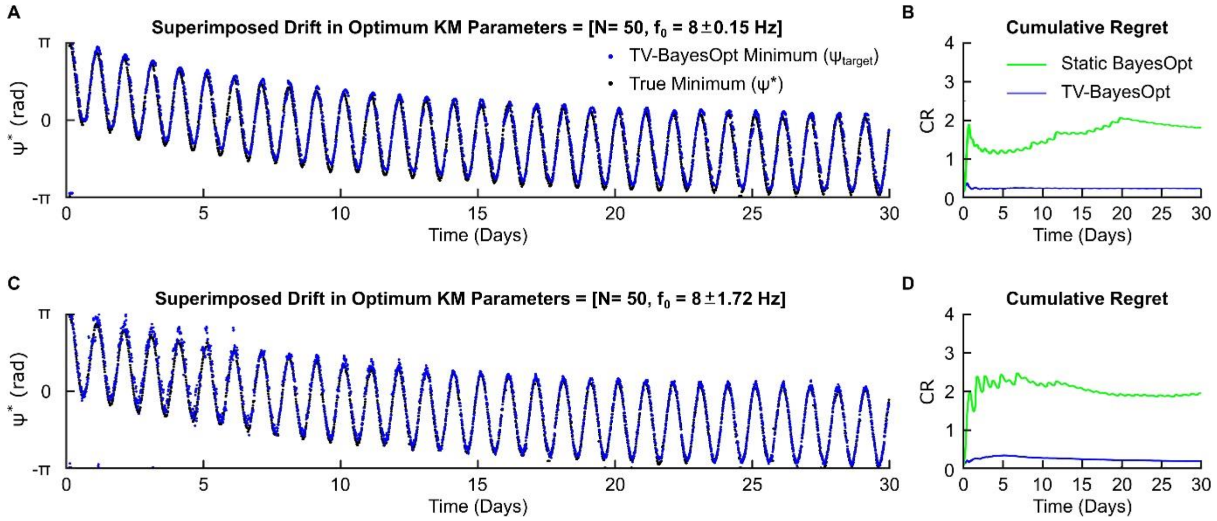

Figure 2: TV-BayesOpt algorithm performance for tracking a superimposed (gradual and periodic) drift in the optimal stimulation phase for phase-locked stimulation, ψ^*, for varying Kuramoto model parameter values. Panels A and C illustrate the tracking performance of the TV-BayesOpt algorithm (blue dots) at locating the true optimal phase value (black dots) for population desynchronization when the Kuramoto model oscillator frequency was centered at 8 Hz. The frequency of oscillators in the underlying population were selected from a Gaussian distribution centered at this frequency. Panel C illustrates that increasing distribution of the oscillator natural frequencies does not affect algorithm performance at tracking the optimum stimulation phase since the shape of the objective is consistent across these parameters. Panels B and D illustrate the associated average regret for the TV-BayesOpt algorithm at tracking the true optimum phase value in comparison to when static BayesOpt was implemented alone for tracking the optimum trajectory in their associated panels A and C.

Moreover, the text below has now been included at lines 154 – 158 to clarify selection of an appropriate stimulation intensity value:

"To simulate effective phase-locked stimulation, I was selected as a stimulation intensity value that resulted in desynchronization of population activity during continuous, 130 Hz high frequency stimulation. In clinical practice identification of an effective stimulation intensity value, or amplitude, is undertaken by systematic, stepwise evaluation of stimulation amplitudes from the clinical parameter space (Volkmann et al., 2002)"

Comment 2:

The authors consider a useful extension of the Kuramoto model to assume two distinct neuron categories based on their phase response curves: type I exclusive delay or advanced spike firing; type II both advance or delay dependent upon specific phase at which stimulation is delivered.

The objective function used in the proposed time-variant Bayesian optimization is the effect of the precise stimulation timing on minimizing the population synchrony. The performance evaluation then computes a cumulative regret using the known true values. This application specific evaluation criteria is arguably a lot more indicative of the practical utility of the proposed approach.

Comment 2 Response:

We thank the reviewer for this suggestion. Lines 424 – 438 in Section 2.4 of the Methods have now been updated to include additional description of the regret calculation that was used to quantify algorithm performance:

"At each optimization step, the associated regret of the algorithm was calculated as

$$R = f(\psi_k^*) - f\left(\psi_{target_k}\right) \qquad (16)$$

Where ψ_k^* is the location of the true optimum value for phase-locked stimulation at optimization step k, ψ_{target_k} is the stimulation phase value tested at the kth optimization step, f(*) is the measured objective function value in response to stimulation at the specified input phase value, i.e. the change in the population mean-phase coherence due to stimulation at the specified input target phase value. Thus, at each optimization step the regret quantified the difference between the suppression achieved by the algorithm in comparison to the maximum possible suppression that was achievable at that optimization step. In a practical implementation this would correspond to the difference between the symptom suppression achieved by the algorithm, objectively quantified based on an accelerometer or LFP power signal at the frequency band of interest recorded from a patient, and the maximum symptom suppression that was possible at that optimization step."

Comment 3:

The optimal phase value for stimulation is considered to be the one leading to the greatest reduction in population synchrony. Some sensitivity analysis is offered to provide intuition in what different phase values are inferred when using alternative covariance structures (e.g. forgetting temporal covariance, periodic covariance), however, it was a bit unclear whether the forgetting term and the temporal period in the proposed forgetting periodic covariance are chosen independently.

Comment 3 Response:

We thank the reviewer for this suggestion. The hyperparameters of the temporal covariance functions utilized by the algorithm were chosen independently. This has now been clarified in Section 2.4 of the updated manuscript at lines 450 - 463:

"For each simulated scenario, the hyperparameters of the utilized temporal covariance functions were independently selected based on the anticipated temporal drift in the data. In this manner, simulations of only gradual or periodic drifts in the phase offset utilized gradual "forgetting" or periodic temporal covariance functions in the algorithm, respectively. Meanwhile in the superimposed drift scenario, the hyperparameters for the gradual "forgetting" and periodic temporal covariance functions were respectively selected based on these anticipated components of the overall simulated drift. For a real-world implementation, longitudinal recordings of biomarker signal(s) (derived from an accelerometer or electrophysiological recordings) should be undertaken prior to algorithm deployment to characterize patient-specific temporal variations (potentially due to the 24-hour circadian rhythm or patient medication cycles). These characterized temporal variations can be subsequently incorporated as a priori information for the algorithm when selecting appropriate hyperparameter values, i.e., the periodic covariance function period."

Comment 4:

It seems to be the case that temporal period has been selected to capture circadian fluctuations (i.e. in 24 hour span) in the particular work, and then a range of values for the forgetting term are offered, but what are the practical trade-offs in achieving tractable inference was a bit unclear.

Comment 4 Response:

We thank the reviewer for this suggestion. The enclosed text has now been added to lines 749 – 754 in section 4.3 of the Discussion to highlight that once the periodic components of the temporal drift have been suitably characterized the practical trade-off of implementing the algorithm with good performance is dependent on selection of a suitable ϵ value to determine the amount of data used for inference:

"Thus, once periodic components of the temporal drift have been suitably characterized the practical trade-off of the algorithm achieving good performance is dependent on identification of an appropriate ϵ value. Selection of an ϵ value lower than necessary will result in the

algorithm utilizing more, potentially redundant data as part of its inference, while too high a value may lead to an insufficient amount of available data for algorithm inference. Selection of an ϵ value thus requires a balance of these two considerations."

Minor Comment 1:

It would be beneficial to comment on the computational consequences in terms of inference which following from replacing the stationarity of the covariance assumption in Nyikosa et al. 2022.

Minor Comment 1 Response:

We thank the reviewer for this suggestion. In Nyikosa et al. 2022 the temporal covariance function is assumed to be stationary. This means that the covariance between two points in space and time depends only on the distance between these points in space and time. The temporal covariance in our approach, however, is not stationary due to our implementation of the periodic covariance function. This means that the covariance between two points in time is no longer dependent solely on the distance between them, but rather on the absolute time at which the points were taken. In this manner, points which were taken at the same phase of the repeating periodic temporal function, i.e., the 24-hour circadian rhythm, will have high covariance, while points taken at opposite phases of the periodic function will have a small covariance. The enclosed text has now been added to lines 712 – 718 of section 4.1 in the updated manuscript to highlight this:

"The temporal covariance in our proposed approach is not stationary due to our implementation of a periodic temporal covariance function. This means that covariance between two sampled points in time is no longer dependent solely on the distance between them, but rather on the absolute time at which the samples were taken. Data points which were sampled at the same phase of a repeating periodic rhythm, i.e., the 24-hour circadian rhythm, will have high covariance, while points taken at opposite phases of the rhythm will have a small covariance."

Minor Comment 2:

The authors did consider variation in the observed and assumed temporal drift, but it would be interesting to see how well the approach works when there are some deviations from the gradual drift assumed by the model in the generated data (e.g. adding different levels of noise during data generation).

Minor Comment 2 Response:

The performance of the algorithm at tracking the optimal stimulation phase in the presence of noise is illustrated below in Figure S1. Zero mean Gaussian distributed noise with a 0.75 or 1.5 standard deviation was added to the superimposed drift trajectory, Figure S1.C, E. The TV-BayesOpt algorithm maintained better performance than static BayesOpt at tracking the noisy drift in the optimum in Figure S1.C as illustrated by the lower cumulative regret value for the algorithm, Figure S1.D. When the standard deviation of the noise was further increased to 1.5 in Figure S1.E clear identification of the optimum trajectory was no longer possible. The algorithm however still maintained improved performance over static BayesOpt at tracking this noisy drift in the optimum, Figure S1.F. This demonstrates the ability of the TV-BayesOpt algorithm to maintain tracking of the structure of the temporal variation of the optimum value in the presence of noise. The below figure is now included as Supplemental Information Figure S1 for the revised manuscript.

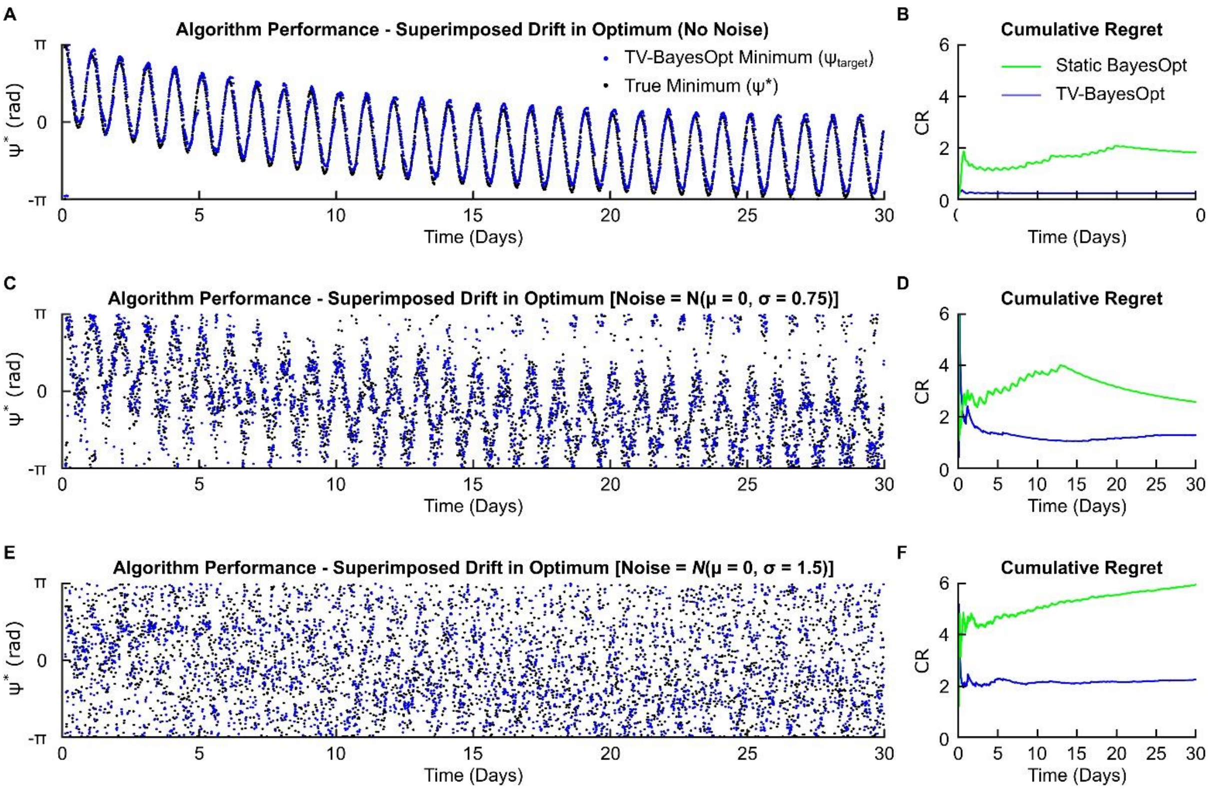

Figure S1: TV-BayesOpt algorithm performance for tracking a superimposed (gradual and periodic) drift in the optimal stimulation phase for phase-locked stimulation, ψ^*, in the presence of noise. Panels A, C and E illustrate the tracking performance of the TV-BayesOpt algorithm (blue dots) at locating the true optimal phase value (black dots) for population desynchronization when no noise, Gaussian distributed noise with zero mean and a 0.75 standard deviation or Gaussian distributed noise with zero mean and a 1.5 standard deviation was added to the simulated optimum trajectory. Panels B, D and F illustrate the associated average regret for the TV-BayesOpt algorithm at tracking the true optimum phase value in comparison to when static BayesOpt was implemented alone for tracking the optimum trajectory in their associated Panels A, C and E.

Minor Comment 3:

Line 150 – 'he' is 'The'

Minor Comment 3 Response:

This has been corrected.

Minor Comment 4:

The kernel equation in Figure 7A seems a bit out of place?

Minor Comment 4 Response:

We thank the reviewer for this suggestion. The temporal kernel equations in Figures 5A, 6A, and 7A of the revised manuscript have been removed. The title of each figure has been updated to highlight the type of drift simulated that was simulated for each corresponding set of simulations.

Reviewer 2

Comment 1:

The results seem solid (I'm not an expert in statistics), but I'm still not convinced that they are generalizible enough to be used for closed loop DBS, as authors claim.

I would like to see if the procedure works for other type of oscillators, or even for spiking neurons, otherwise the work seems too theoretical.

Comment 1 Response:

We thank the reviewer for this suggestion. To explore the generalizability of our approach, we interfaced the algorithm with a computational model of the motor cortex microcircuit (MCM) during phase-locked stimulation. This model was previously used in (West et al., 2023) to explore variations in 20 Hz oscillations in the primary motor cortex during different movement states. The model architecture follows the laminar structure of the motor cortex incorporating deep, middle, and superficial pyramidal layers, as well as local and global inhibition, depicted in Figure 3.

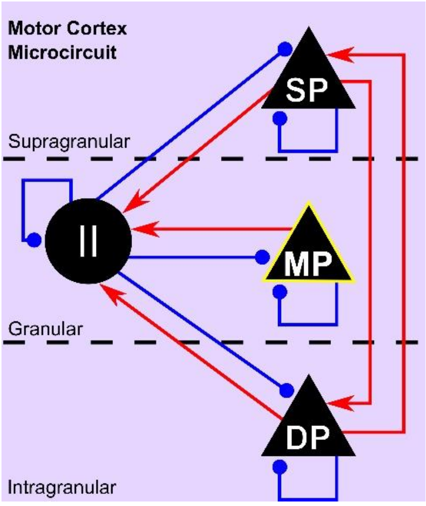

Figure 3: Overview of Motor Cortex Microcircuit Model. The model is laminarly structured with a supragranular, granular and infragranular layers. The activity of each population is represented as a Wilson-Cowan population firing rate model. Inhibitory and excitatory synaptic connections are represented as blue and red arrows, respectively.

Neuronal dynamics are described by coupled Wilson-Cowan equations in which the average firing rate of each cell population (middle *MP*, superficial *SP*, inhibitory interneuron *II*, deep *DP*) is described by the following state equations:

$$\frac{dR_{MP}}{dt} = \frac{1}{T_{MP}}\left(-R_{MP} + S(\{-G_{MP\to MP}R_{MP} - G_{II\to MP}R_{II} + \ G_{SP\to MP}R_{SP}\}, M_{MP}, S_{MP}, B_{MP})\right) \quad (1)$$

$$\frac{dR_{SP}}{dt} = \frac{1}{T_{SP}}\left(-R_{SP} + S(\{-G_{SP\to SP}R_{SP} + G_{MP\to SP}R_{MP} - G_{II\to SP}R_{II} + G_{DP\to SP}R_{DP}\}, M_{SP}, S_{SP}, B_{SP})\right) \quad (2)$$

$$\frac{dR_{II}}{dt} = \frac{1}{T_{II}}\left(-R_{II} + S(\{-G_{II\to II}R_{II} + G_{MP\to II}R_{MP} + G_{DP\to II}R_{DP} + G_{SP\to II}R_{SP}\}, M_{II}, S_{II}, B_{II})\right) \quad (3)$$

$$\frac{dR_{DP}}{dt} = \frac{1}{T_{DP}}\left(-R_{DP} + S(\{-G_{DP\to DP}R_{DP} - G_{II\to DP}R_{II} + G_{SP\to DP}R_{SP}\}, M_{DP}, S_{DP}, B_{DP})\right) \quad (4)$$

where *T* is the population time constant, *G* is the weight of the delayed synaptic coupling between populations, and *S(I, M, S, B)* is the sigmoidal transfer function for the total input *I* to each population given within the curly brackets:

$$S(I, M, S, B) = \frac{M}{1 + exp^{\frac{-S\,I}{M}} \cdot \frac{(M - B)}{B}} \quad (5)$$

where *M* reflects the maximum firing rate, *S* the slope of the sigmoid, and *B* the spontaneous baseline firing rate for the population. Further information on the parameter values used in this model can be found in Supplementary Table 1 of (West et al., 2023).

We set up a model of phase locked stimulation in this system, that could reflect a closed-loop stimulation protocol. We chose to stimulate deep cell layers, whilst sensing from the superficial layers. At baseline, i.e., when no stimulation was applied, there was elevated beta-band activity (14 – 21 Hz) in the superficial layer. When phase-locked stimulation was applied to the deep layer, beta-band activity in the superficial population was either amplified or suppressed, depending upon the phase that stimulation was delivered, Figure 4. The change in relative beta-band power from baseline was set as the objective function for our TV-BayesOpt algorithm to optimize the target phase for stimulation. Please note that this is equivalent to the ARC for the Kuramoto population, described in the main text of the manuscript.

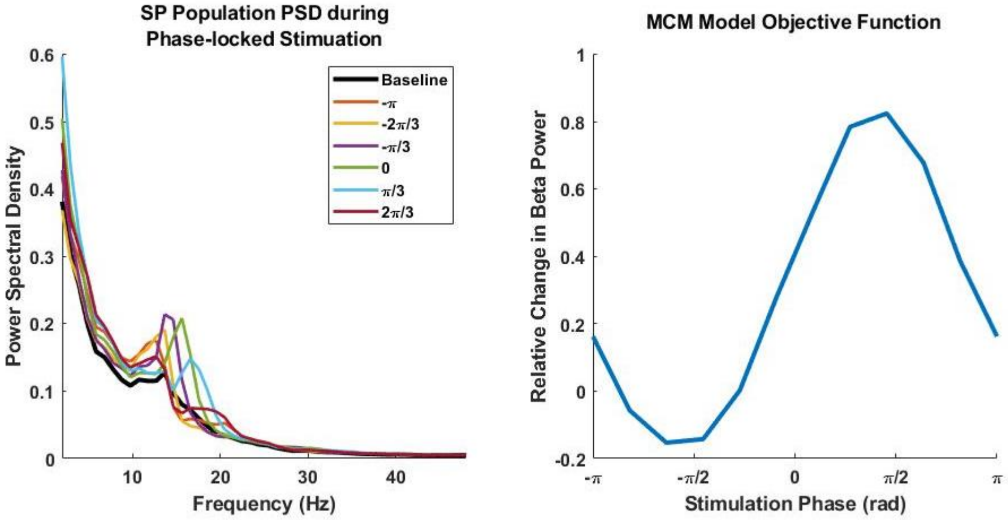

Figure 4: Power spectral density and relative change in beta-band power during phase-locked stimulation. The left panel represents the distribution of power of the SP population at baseline (when no stimulation is applied) and during phase-locked stimulation of the DP population at specific target phases for stimulation. The right panel illustrates the relative change in beta-band power during phase-locked stimulation. This objective function is equivalent to the ARC of the Kuramoto population in the manuscript.

To simulate a temporal drift, the inhibitory drive to the deep cell population was modulated by varying the strength of the synaptic connection from the global inhibitory cell layer (II) to the deep cell layer. This resulted in a periodic temporal drift in the objective function which was used to test the performance and generalizability of the TV-BayesOpt algorithm. The left panel of Figure 5 illustrates the capability of the TV-BayesOpt algorithm to track the variation in the optimum stimulation phase in the objective function over the optimization process. The cumulative regret of the algorithm was less than that of static BayesOpt as illustrated by the right panel in Figure 5 below. This highlights the improved performance of the algorithm at tracking a dynamically varying optimum.

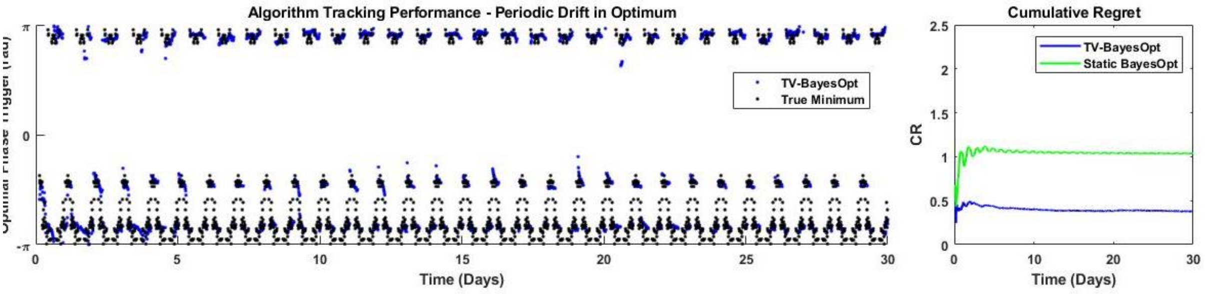

Figure 5:TV-BayesOpt algorithm performance for tracking a periodic drift in the optimal stimulation phase for phase-locked stimulation, $\boldsymbol{\psi}^{*}$, for varying inhibitory input to the DP population in the MCM model. The left panel illustrates the tracking performance of the TV-BayesOpt algorithm (blue dots) at locating the true optimal phase value (black dots) for desynchronizing beta-band oscillatory activity measured in the SP population. The right panel illustrates the associated average regret for the TV-BayesOpt algorithm at tracking the true optimum phase value in comparison to when static BayesOpt was implemented alone for tracking the optimum trajectory.

Here and in the main manuscript we demonstrated the utility of the TV-BayesOpt algorithm using two specific examples involving optimization of stimulation phase in either Kuramoto oscillators at tremor frequencies (8 Hz) or coupled Wilson-Cowan equations modelling the behavior of the motor cortex microcircuit at the beta band (14-21 Hz). It should be noted that this method could be readily adapted for other parameters and/or systems. Alternative stimulation parameters such as the stimulation amplitude, frequency or pulse duration may be optimized. Similarly, the biomarker used to derive the objective function does not need to be rhythmic, it could vary in response to the stimulation parameter that is being optimized. We have however focused on the use of a rhythmic biomarker in the manuscript as there is ample evidence that oscillatory neural activity can vary at multiple timescales due to nesting of biological rhythms that are likely to modify the efficacy of neuromodulation therapies over long time spans (Fleming et al., 2022; van Rheede et al., 2022).

Comment 2:

Similarly, it is not clear what is the reason for the choice of natural frequency, or why the number of oscillators is chosen to be so low.

Comment 2 Response:

We thank the reviewer for this suggestion. The natural frequency of the oscillators was selected as 8 Hz to simulate oscillatory activity characteristic of Essential Tremor. This is now clarified at lines 127 – 129 in the Methods section 2.2 of the revised manuscript with the below text:

"In this study, to model synchronous oscillatory activity characteristic of Essential Tremor, where tremor is observed to occur between 4 – 12 Hz, ω was selected as 8 Hz (Thanvi et al., 2006)."

The Kuramoto model was chosen to explore the performance of the TV-BayesOpt algorithm since model parameters can be tuned to represent pathologically increased synchronization of neural rhythms at a variety of frequency bands. Figure 6 below illustrates the Kuramoto model parameter space (the coupling strength between oscillators in the population vs. the standard deviation of oscillator frequencies selected from a Gaussian distribution centered around a natural frequency) for a population of oscillators with a natural frequency of either 8 and 23 Hz, representing Essential tremor (Thanvi et al., 2006) and Parkinson's disease (Kühn et al., 2006), respectively. These oscillopathies are based on extensive literature highlighting elevated

rhythmic activity patterns at these frequency bands and correspond to regions of the Kuramoto parameter space where the population is synchronized, Figure 6.

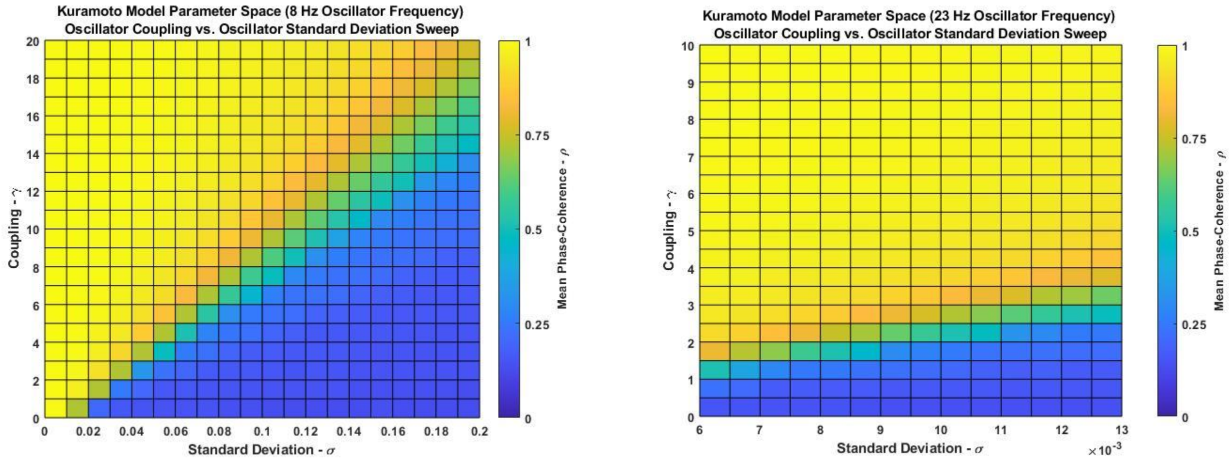

Figure 6: Average population synchrony of Kuramoto oscillators as a function of population coupling strength and distribution of oscillator frequencies from population center frequency. The left panel illustrates the parameter space for a population of Kuramoto oscillators whose natural frequency is centered at 8 Hz, while the right panel demonstrates the same for a population centered on 23 Hz. Yellow regions in the parameter space indicate parameter values that result in high population synchrony and a well-defined objective function for the TV-BayesOpt algorithm. Blue regions of the parameter space in contrast correspond to parameter values that produce low population synchrony where the objective function is not well-defined.

When the oscillator population is sufficiently synchronized the shape of the objective function (the ARC) for the algorithm is not affected by the frequency, standard deviation or number of oscillators in the underlying population. This is illustrated below in Figure S2. Panels A and B of Figure S2 represent the performance of the TV-BayesOpt algorithm at tracking a superimposed (gradual and periodic) drift in the optimum stimulation phase for a population of 50 oscillators with natural frequencies 8 ± 0.15 Hz as reported in Figure 7 of the main manuscript. In Figure S2.C,D the distribution of natural frequencies for the population was increased to 8 ± 1.72 Hz, while in Figure S2.E,F the size of the population was increased to 100 with the distribution of natural frequencies being kept at 8 ± 0.15 Hz. In both cases, the distribution of natural frequencies and size of the population did not affect the performance of the algorithm as demonstrated by the similar cumulative regret traces in panels B, D and F. Similarly, increasing the natural frequency of the population to 23 ± 1.85 Hz did not affect the performance of the algorithm as highlighted in Figure S2.G, H. The algorithm performance is not influenced by the underlying parameters of the population because the shape of the objective function remains the same across the varying population parameters. Figure S2 is now included as Supplemental Information for the revised manuscript.

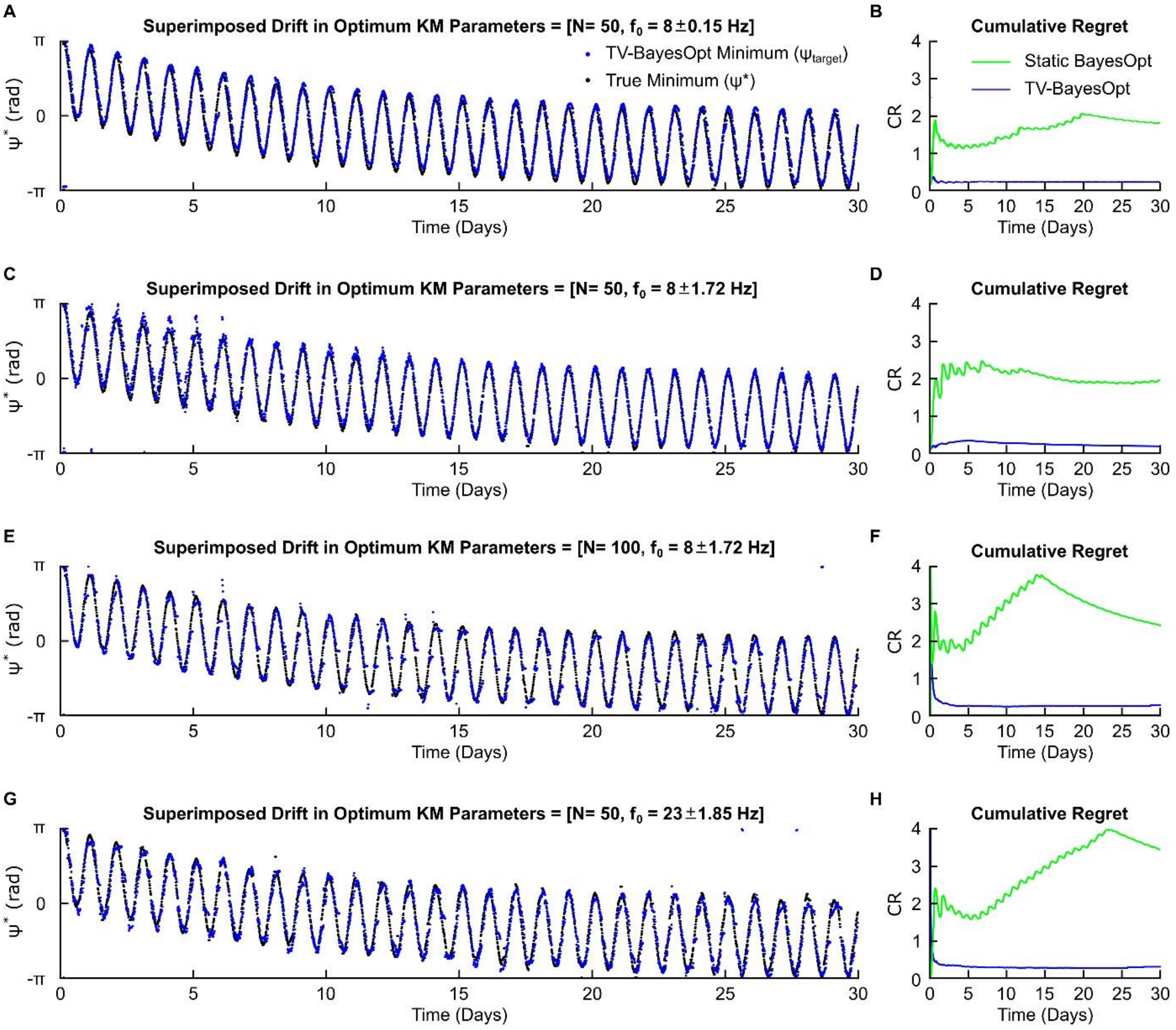

Figure S2: TV-BayesOpt algorithm performance for tracking a superimposed (gradual and periodic) drift in the optimal stimulation phase for phase-locked stimulation, ψ^*, for varying Kuramoto model parameter values. Panels A, C, E and G illustrate the tracking performance of the TV-BayesOpt algorithm (blue dots) at locating the true optimal phase value (black dots) for population desynchronization when the Kuramoto model oscillator frequency was centered at 8 or 23 Hz. The frequency of oscillators in the underlying population were selected from a Gaussian distribution centered around each frequency. Panel C, E and G illustrate that increasing distribution of the oscillator natural frequencies, the size of the population or the natural frequency of the oscillator population does not affect algorithm performance at tracking the optimum stimulation phase since the shape of the objective is consistent across these parameters. Panels B, D, F and H illustrate the associated average regret for the TV-BayesOpt algorithm at tracking the true optimum phase value in comparison to when static BayesOpt was implemented alone for tracking the optimum trajectory in their associated panels A, C, E and G.

Comment 3:

In the same sense, I'd imagine that the result of the model would be more realistic if there is also noise and even distribution of natural frequencies.

Comment 3 Response:

We thank the reviewer for this suggestion. As described in our response to comment 2 above the algorithm maintained its performance when the distribution of natural frequencies in the population was increased, Figure S2.C, D.

To further explore the performance of the algorithm in the presence of noise we simulated superimposed (gradual and periodic) temporal drifts in the optimum stimulation value with zero mean Gaussian distributed noise added to the simulated optimum trajectory, Figure S1. When the Gaussian distributed noise was simulated with a standard deviation of 0.75 the TV-BayesOpt algorithm was able to maintain its improved performance at tracking the stimulation optimum when compared to static BayesOpt, Figure S1.C,D. When the standard deviation of the noise was increased to 1.5, the optimum trajectory could no longer be clearly identified, Figure S1.E. In this case, the algorithm still outperformed the static BayesOpt algorithm (Figure S1.F) but its performance was greatly reduced from the case when no noise was present, Figure S1.B. The below figure is now included as Supplemental Information Figure S1 for the revised manuscript.

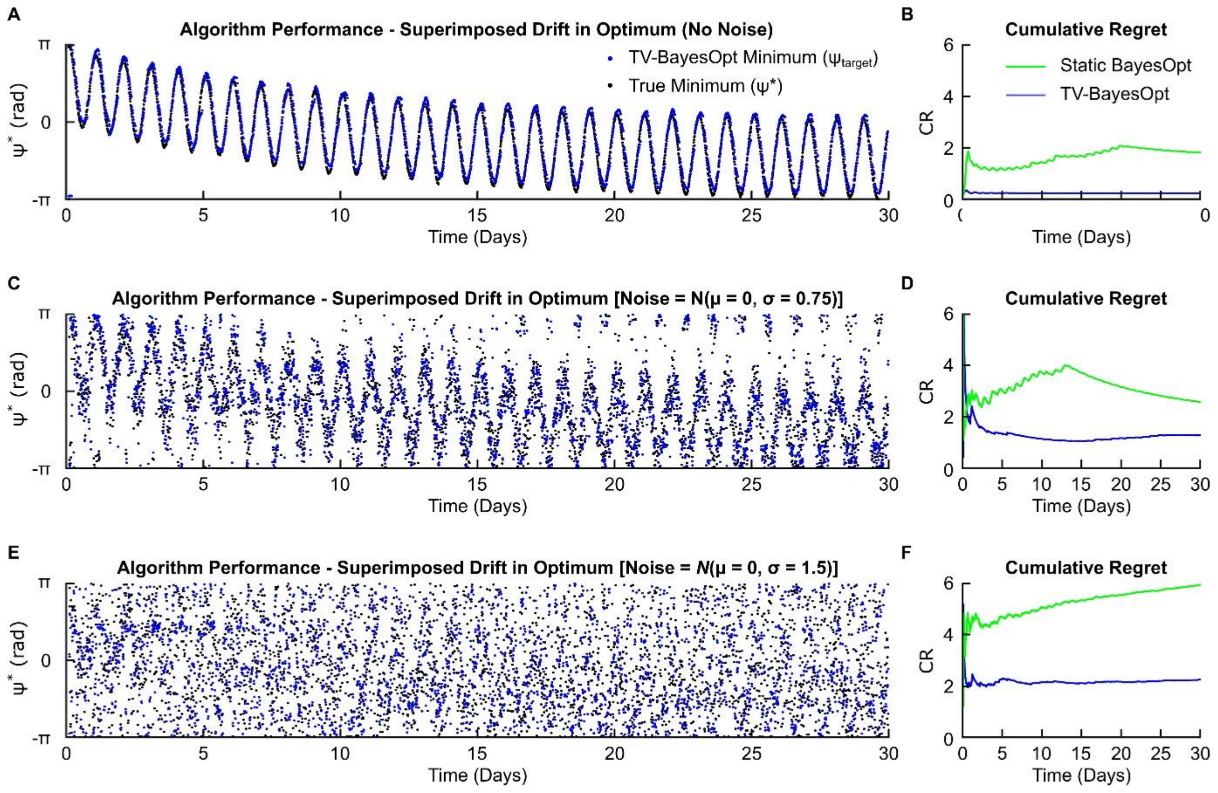

Figure S1: TV-BayesOpt algorithm performance for tracking a superimposed (gradual and periodic) drift in the optimal stimulation phase for phase-locked stimulation, ψ^*, in the presence of noise. Panels A, C and E illustrate the tracking performance of the TV-BayesOpt algorithm (blue dots) at locating the true optimal phase value (black dots) for population desynchronization when no noise, Gaussian distributed noise with zero mean and a 0.75 standard deviation or Gaussian distributed noise with zero mean and a 1.5 standard deviation was added to the simulated optimum trajectory. Panels B, D and F illustrate the associated average regret for the TV-BayesOpt algorithm at tracking the true optimum phase value in comparison to when static BayesOpt was implemented alone for tracking the optimum trajectory in their associated Panels A, C and E.

Finally, to ensure that the population was not already synchronized at initiation of the simulations the initial phase of each oscillator in the population was selected from a uniform distribution between [0, 2π]. This ensured that the population was desynchronized at the beginning of the simulation. This is now clarified by the inclusion of the below text at lines 385 -386 in section 2.4 of the revised manuscript:

"The initial phase of each oscillator in the population was selected from a uniform distribution between [0, 2π] at the start of the simulation."

Minor Comment 1:

It is confusing that both spatial covariance and coupling are represented with K.

Minor Comment 1 Response:

Additional subscripts 's' and 't' have been included in the updated manuscript to clarify whether a covariance function is a spatial covariance function (K_s) or a temporal covariance function (K_t), respectively. Moreover, the symbol used as the coupling term for the Kuramoto Model population has been updated from 'K' to 'γ' in the revised manuscript for clarity.

Minor Comment 2:
Kuramoto order parameter is not defined.

Minor Comment 2 Response:
Reference to the Kuramoto model "order parameter" has been updated to the population "mean phase-coherence" throughout the updated manuscript to more accurately describe the average population synchrony that is quantified during each step of the optimization process.

Minor Comment 3:
why K and omega have subscripts biomarker, when biomarker is a measurable quantity, such as rho, while coupling and the natural frequency aren't.

Minor Comment 3 Response:
The subscript 'biomarker' has been removed from the natural frequency (ω) and coupling (γ) terms in the description of the Kuramoto model in the revised manuscript.

Minor Comment 4:
the representation of the periodic covariance function is also not typical

Minor Comment 4 Response:
The periodic covariance function included in the manuscript is an exponential sine squared kernel. We utilized the general formalism for this covariance function as reported in (Pearce et al., 2020):

$$K(x, x') = \sigma^2 \exp\left(-\frac{2\sin^2(\frac{\pi(x - x')}{p})}{l^2} \right)$$

where l is the length scale, σ^2 is a scaling parameter, p is the period over which the function repeats, and x and x' are two arbitrary data points from the parameter space. The representation of the periodic covariance kernels in the manuscript are

$$K_s(x,x') = \exp\left(-\frac{2\sin^2\left(\frac{\pi|x-x'|}{T_x}\right)}{{l_x}^2}\right)$$

for the spatial kernel where σ^2 is assumed to be 1, l_x is the length scale, and T_x is the function's (spatial) period. Similarly, the temporal covariance kernel is represented as

$$K_t(t,t') = \exp\left(-\frac{2\sin^2\left(\frac{\pi|t-t'|}{T_t}\right)}{{l_{t,p}}^2}\right)$$

where σ^2 is assumed to be 1, $l_{t,p}$ is the length scale, and T_t is the function's (temporal) period.

The authors are very happy to amend this further in the revised manuscript if the reviewer could please specify their particular concerns regarding the notation.

Minor Comment 5:

maybe it is worth mentioning in the discussion that there is already some analytical results about Kuramoto model with time-varying parameters. Similarly, the authors should specify that their algorithm is supposed to work only for slow-forcing of the coupling, when adiabatic reduction is also possible. The algorithm would probably fail for fast modulation of K.

Minor Comment 5 Response:

We thank the reviewer for this suggestion. The following text has been added to lines 665 – 671 of the Discussion section of the revised manuscript to highlight previous literature which has investigated time variations in the Kuramoto model behavior using time-dependent parameters:

"We explored the efficacy of the proposed approach in the Kuramoto Model which is a well-established mathematical model that has been extensively used in literature to investigate synchronization in biological systems. The model has been previously used to simulate time variability in systems using time-dependent model parameters (Cumin and Unsworth, 2007; Petkoski and Stefanovska, 2012), in addition to modelling the effects of electrical stimulation therapy on neural synchrony (Weerasinghe et al., 2019). In this current study, slow variations in therapy efficacy were emulated by incremental the shape of the model population PRC overtime."

Line 31 of the abstract in the revised manuscript has been updated to clarify that the performance of the algorithm presented in this study was investigated only for tracking slow

variations over time. Additionally, the below text has been incorporated at lines 777 – 781 of the Discussion section 4.2 to highlight that this work only explored slow temporal variations in algorithm performance:

"Most notably, only relatively slow variations in the optimum stimulation parameter set were investigated in this present study to emulate temporal variations associated with biological rhythmicity and disease progression. The performance of the algorithm to track fast changes in the optimum parameter set which may be encountered during different activities of daily living or contexts was not evaluated."

Relevant References:

Cumin, D., Unsworth, C.P., 2007. Generalising the Kuramoto model for the study of neuronal synchronisation in the brain. Phys. Nonlinear Phenom. 226, 181–196. https://doi.org/10.1016/j.physd.2006.12.004

Fleming, J.E., Kremen, V., Gilron, R., Gregg, N.M., Zamora, M., Dijk, D.J., Starr, P.A., Worrell, G.A., Little, S., Denison, T.J., 2022. Embedding digital chronotherapy into bioelectronic medicines. iScience 25, 104028–104028. https://doi.org/10.1016/J.ISCI.2022.104028

Kühn, A.A., Kupsch, A., Schneider, G.H., Brown, P., 2006. Reduction in subthalamic 8-35 Hz oscillatory activity correlates with clinical improvement in Parkinson's disease. Eur. J. Neurosci. 23, 1956–1960. https://doi.org/10.1111/j.1460-9568.2006.04717.x

Pearce, T., Tsuchida, R., Zaki, M., Brintrup, A., Neely, A., 2020. Expressive Priors in Bayesian Neural Networks: Kernel Combinations and Periodic Functions, in: Proceedings of The 35th Uncertainty in Artificial Intelligence Conference. Presented at the Uncertainty in Artificial Intelligence, PMLR, pp. 134–144.

Petkoski, S., Stefanovska, A., 2012. Kuramoto model with time-varying parameters. Phys. Rev. E 86, 046212. https://doi.org/10.1103/PhysRevE.86.046212

Thanvi, B., Lo, N., Robinson, T., 2006. Essential tremor—the most common movement disorder in older people. Age Ageing 35, 344–349. https://doi.org/10.1093/ageing/afj072

van Rheede, J.J., Feldmann, L.K., Busch, J.L., Fleming, J.E., Mathiopoulou, V., Denison, T., Sharott, A., Kühn, A.A., 2022. Diurnal modulation of subthalamic beta oscillatory power in Parkinson's disease patients during deep brain stimulation. Npj Park. Dis. 2022 81 8, 1–12. https://doi.org/10.1038/s41531-022-00350-7

Volkmann, J., Herzog, J., Kopper, F., Deuschl, G., 2002. Introduction to the programming of deep brain stimulators. Mov. Disord. Off. J. Mov. Disord. Soc. 17 Suppl 3, S181-187. https://doi.org/10.1002/mds.10162

Weerasinghe, G., Duchet, B., Cagnan, H., Brown, P., Bick, C., Bogacz, R., 2019. Predicting the effects of deep brain stimulation using a reduced coupled oscillator model. PLOS Comput. Biol. 15, e1006575–e1006575. https://doi.org/10.1371/JOURNAL.PCBI.1006575

West, T.O., Duchet, B., Farmer, S.F., Friston, K.J., Cagnan, H., 2023. When do bursts matter in the primary motor cortex? Investigating changes in the intermittencies of beta rhythms associated with movement states. Prog. Neurobiol. 221, 102397. https://doi.org/10.1016/j.pneurobio.2022.102397
